# Metabolite Biomarkers for Early Detection of Pancreatic Ductal Adenocarcinoma: A Systematic Review

**DOI:** 10.7759/cureus.74528

**Published:** 2024-11-26

**Authors:** Anthony Eze-odurukwe, Abdur Rehman, Lois Ayinla, Nabila N Anika, Ramsha Shahid, Amarachi L Ugwuoru, Muzafar Mansoor, Muhammad Kamran

**Affiliations:** 1 Surgery, Salford Royal NHS Foundation Trust, Manchester, GBR; 2 Surgery, Mayo Hospital, Lahore, PAK; 3 General Medicine, All Saints University School of Medicine, Roseau, DMA; 4 Surgery, Baylor College of Medicine, Houston, USA; 5 Medicine and Surgery, Holy Family Red Crescent Medical College and Hospital, Dhaka, BGD; 6 Physiology, Akhtar Saeed Medical and Dental College, Islamabad, PAK; 7 Public Health, Ebonyi State University, Abakaliki, NGA; 8 Internal Medicine, Allama Iqbal Medical College, Lahore, PAK; 9 Internal Medicine, Mayo Hospital, Lahore, PAK

**Keywords:** biomarkers, cancer screening, early detection, metabolomics, pancreatic ductal adenocarcinoma

## Abstract

Pancreatic ductal adenocarcinoma (PDAC) remains one of the most lethal malignancies, with a poor prognosis. This poor prognosis is largely attributed to a late-stage diagnosis. Recent advancements in metabolomics have emerged as a promising avenue for biomarker discovery in PDAC. This systematic review evaluates the potential of metabolite biomarkers for early detection of PDAC. Four studies meeting the inclusion criteria were analyzed, encompassing experimental, case-control, and prospective cohort designs. Key findings include the identification of distinct metabolic subtypes in PDAC with varying sensitivities to metabolic inhibitors. A biomarker signature comprising nine metabolites plus CA19-9 showed high accuracy in distinguishing PDAC from chronic pancreatitis, outperforming CA19-9 alone. Another study identified a five-metabolite signature demonstrating high diagnostic accuracy for pancreatic cancer, differentiating it from type 2 diabetes mellitus. A two-metabolite model (isoleucine and adrenic acid) showed superior performance in detecting stage-I PDAC compared to CA19-9. These studies consistently demonstrate altered metabolic pathways in PDAC patients compared to healthy controls and those with benign pancreatic conditions. Integrating metabolomic data with other molecular profiling approaches has become a powerful strategy for improving diagnostic accuracy. However, challenges remain, including the influence of confounding factors, the need for large-scale validation studies, and the standardization of metabolomic methods. The potential of artificial intelligence in interpreting complex metabolomic data offers promising avenues for future research. This review highlights the significant potential of metabolite biomarkers in early PDAC detection while emphasizing the need for further validation and refinement of these approaches.

## Introduction and background

Pancreatic ductal adenocarcinoma (PDAC) remains one of the most lethal malignancies, with a dismal five-year survival rate of less than 10% [1]. This poor prognosis is largely attributed to the late-stage diagnosis of the disease, as early-stage PDAC is often asymptomatic or presents with vague, nonspecific symptoms. By the time most cases are detected, the cancer has typically progressed to an advanced stage, limiting treatment options and effectiveness. Therefore, there is an urgent need for reliable early detection methods to improve patient outcomes and survival rates.

In recent years, the field of metabolomics has emerged as a promising avenue for biomarker discovery in various cancers, including PDAC [2]. Metabolomics involves the comprehensive study of small molecule metabolites in biological systems, providing a snapshot of cellular processes and physiological states. The metabolome is highly sensitive to pathological changes, making it an ideal target for identifying potential biomarkers of early-stage cancer [3].

Several studies have explored the use of metabolite biomarkers for early PDAC detection, focusing on various biological fluids such as blood, urine, and pancreatic juice. These investigations have revealed altered metabolic profiles in PDAC patients compared to healthy controls and those with benign pancreatic conditions. For instance, Mayerle et al. [4] identified a plasma metabolite signature that could distinguish PDAC from chronic pancreatitis and healthy controls with high accuracy. Similarly, Fukutake et al. [5] reported a urinary metabolite panel capable of detecting early-stage PDAC with promising sensitivity and specificity.

The potential advantages of metabolite biomarkers for early PDAC detection are numerous. Firstly, metabolites represent the end products of cellular processes, reflecting the overall physiological state more directly than genomic or proteomic markers. Secondly, metabolomic profiling can be performed on easily accessible biological fluids, enabling non-invasive screening [6]. Thirdly, advances in analytical technologies, such as mass spectrometry and nuclear magnetic resonance spectroscopy, have significantly improved the sensitivity and specificity of metabolite detection.

Despite these promising developments, the field of metabolite biomarkers for early PDAC detection faces several challenges. These include the heterogeneity of PDAC tumors, the influence of confounding factors such as diet and medication on metabolomic profiles, and the need for large-scale validation studies. Additionally, the integration of metabolomic data with other molecular profiling approaches, such as genomics and proteomics, may be necessary to develop robust, multi-modal early detection strategies [7].

Given the rapidly evolving nature of this field and its potential impact on PDAC management, a comprehensive review of the current literature is warranted. This systematic review aims to evaluate the potential of metabolite biomarkers in the early detection of PDAC by synthesizing and critically appraising the available evidence. We will assess the diagnostic performance of various metabolite biomarkers, examine the analytical platforms and methodologies employed, and identify knowledge gaps and future research directions. By consolidating the current state of knowledge on metabolite biomarkers for early PDAC detection, this review seeks to inform future research efforts and contribute to the development of effective screening strategies. Ultimately, the goal is to improve early diagnosis rates and, consequently, the prognosis for patients with this devastating disease.

## Review

Materials and methods

This systematic review is designed and reported following PRISMA (Preferred Reporting Items for Systematic Reviews and Meta-Analyses) and AMSTAR (Assessing the Methodological Quality of Systematic Reviews) guidelines, ensuring a methodologically rigorous and comprehensive evaluation of the included studies.

Search Strategy

A thorough and comprehensive literature search was performed in March 2024 across several electronic databases, including PubMed, the Cochrane Central Register of Controlled Trials (CENTRAL), and ScienceDirect. The search strategy was meticulously crafted in collaboration with a medical librarian, utilizing a combination of relevant keywords and subject headings pertinent to "metabolite biomarkers," "early detection," and "pancreatic ductal adenocarcinoma." These terms were chosen to encapsulate the primary focus of the review, ensuring that studies exploring biomarkers for the early detection of PDAC were comprehensively identified. The search was limited to studies published from the inception of the respective databases up until February 2024 to include a broad range of historical and contemporary research. Additionally, manual searches of the reference lists of relevant systematic reviews and the included studies were conducted to identify further pertinent studies that may have been overlooked in the initial database search.

Eligibility Criteria

To ensure relevance and quality, studies were deemed eligible for inclusion if they comprised original research utilizing observational (such as cohort and case-control) or experimental (including randomized controlled trials and quasi-experimental designs) methodologies. The focus was on studies involving patients diagnosed with PDAC. Crucially, eligible studies are needed to assess metabolites as potential biomarkers for the early detection of this type of cancer. Studies were required to report quantitative data demonstrating the association between metabolite biomarkers and early detection of PDAC. Studies were excluded if they concentrated on biomarkers for conditions other than PDAC, were limited to animal models without corresponding human data, or were categorized as case reports, reviews, editorials, or commentaries. Furthermore, only studies published in English were considered to maintain consistency in the language of the research being reviewed.

Study Selection

The process of study selection was rigorous and systematic. Initially, two independent reviewers screened the titles and abstracts of all records retrieved from the literature search to identify potentially eligible studies. This initial screening was critical in filtering out studies that clearly did not meet the eligibility criteria. Following this, full-text versions of all potentially relevant records were obtained and subjected to a detailed assessment by the same two reviewers. Each study was carefully evaluated against the predefined eligibility criteria. Any discrepancies between the reviewers regarding study inclusion were resolved through discussion and, if necessary, by consulting a third reviewer to reach a consensus. This multi-step selection process was designed to minimize bias and ensure that only the most relevant and high-quality studies were included in the review.

Data Extraction

Data extraction was carried out systematically to ensure the consistency and accuracy of the information gathered from each included study. One reviewer used a standardized data extraction form to collect detailed information from each study, including study characteristics (such as author, year of publication, country, and study design), biomarkers studied, and reported outcomes. This form was designed to capture all relevant data necessary for a thorough analysis of the included studies. After the initial extraction, a second reviewer checked the extracted data for accuracy and completeness, ensuring that any discrepancies or errors were identified and corrected. This dual-review process was implemented to enhance the reliability of the data extraction and to ensure that the synthesis of the studies would be based on accurate and comprehensive data. By adhering to these rigorous methods, this systematic review aims to provide a robust and comprehensive synthesis of the evidence on metabolite biomarkers for the early detection of PDAC.

Results

Study Selection Process

**Figure 1:**
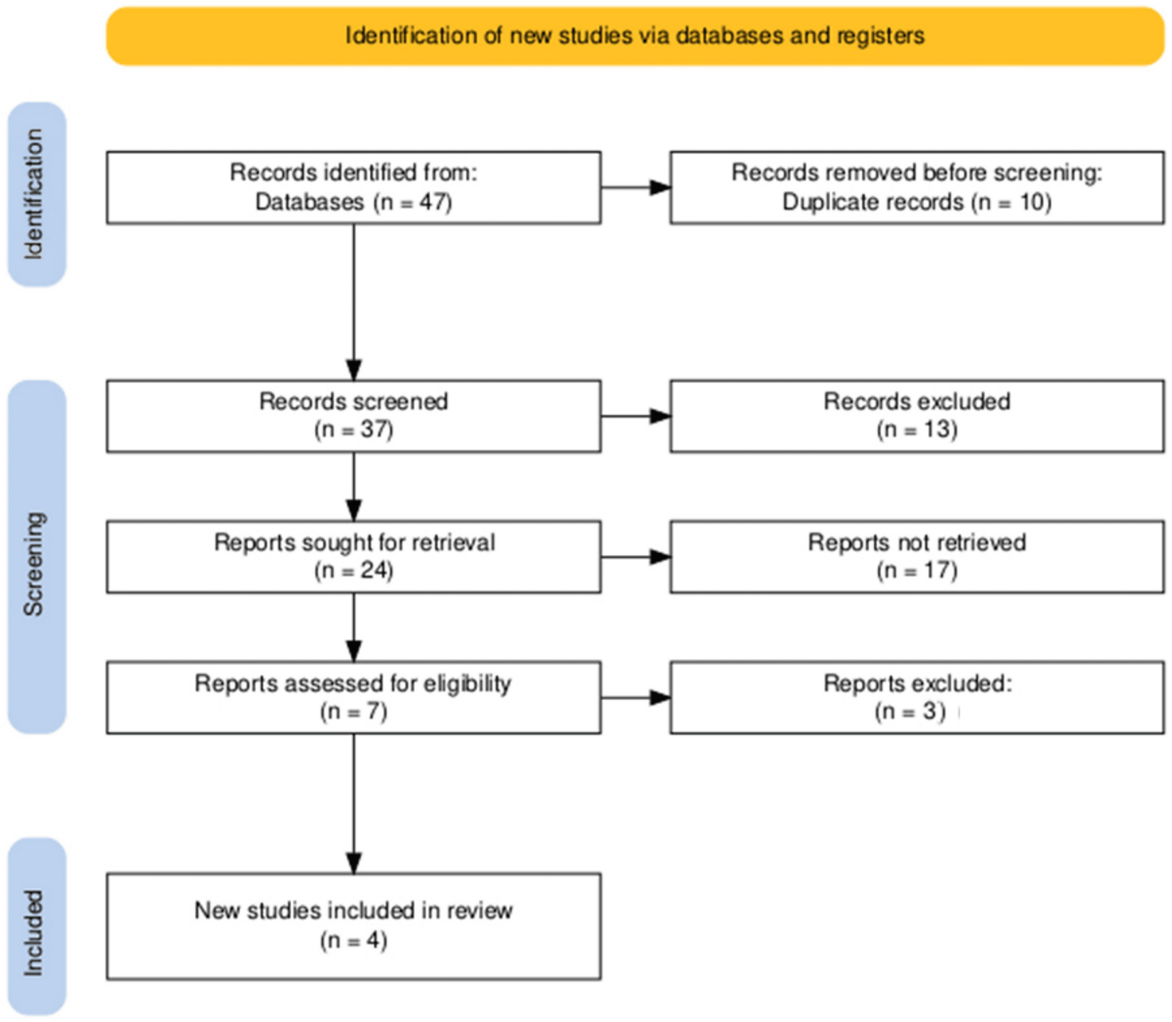
PRISMA diagram illustrating the study selection process. PRISMA: Preferred Reporting Items for Systematic Reviews and Meta-Analyses

Our comprehensive database search initially identified 47 articles. After removing 10 duplicates, we screened the titles and abstracts of the remaining 37 publications. This initial screening led to the selection of seven potentially relevant studies, which underwent full-text review for eligibility assessment. Following this thorough evaluation, four articles met our predefined inclusion criteria and were included in the final analysis. A manual search of the reference lists of these selected articles did not yield any additional eligible studies. The entire selection process is illustrated in the PRISMA flowchart (Figure 1).

Study Characteristics

This systematic review included four studies that investigated metabolite biomarkers for the early detection or characterization of PDAC. The studies were published between 2015 and 2022 in high-impact journals, including Proceedings of the National Academy of Sciences (PNAS), Gut, International Journal of Endocrinology, and Frontiers in Oncology. The research designs varied across studies, encompassing experimental, case-control, and prospective cohort approaches (Table 1).

**Table 1:** Summary of the main findings of the included studies. PDAC: Pancreatic ductal adenocarcinoma; CP: chronic pancreatitis; PC: pancreatic cancer; DM: diabetes mellitus; lysoPC: lysophosphatidylcholine

Author	Study Title	Journal	Year	Study Type	Sample Size	Biomarker(s) Studied	Main Findings
Daemen et al. [8]	Metabolite profiling stratifies pancreatic ductal adenocarcinomas into subtypes with distinct sensitivities to metabolic inhibitors	Proceedings of the National Academy of Sciences (PNAS)	2015	Experimental	38	Twenty-hundred and fifty-six metabolites, gene expression	Identified three metabolic subtypes in PDAC: slow proliferating, glycolytic, and lipogenic. The glycolytic subtype was sensitive to inhibitors of glycolysis, glutamine metabolism, and redox balance. The lipogenic subtype was sensitive to inhibitors of lipid synthesis. Glycolytic subtype associated with mesenchymal features, lipogenic with epithelial features. Metabolic subtypes and sensitivities to metabolic inhibitors validated in ~200 non-PDAC cell lines.
Mayerle et al. [4]	Metabolic biomarker signature to differentiate pancreatic ductal adenocarcinoma from chronic pancreatitis	Gut	2017	Case-control	914 (271 with PDAC, 282 with CP, 100 with liver cirrhosis, 261 healthy and non-pancreatic disease controls)	Metabolomic profiles of 477 metabolites identified by gas chromatography–mass spectrometry and liquid chromatography–tandem mass spectrometry. The final biomarker signature included nine metabolites plus CA19-9	The biomarker signature distinguished PDAC from CP with high accuracy: (a) Training set: AUC 0.96, sensitivity 94.9%, specificity 85% (b) Test set: AUC 0.94, sensitivity 89.9%, specificity 91.3%. Performed significantly better than CA19-9 alone. Detected 98% of resectable PCs. Reduced false positives from 23% with CA19-9 to 9% with the biomarker signature. Reduced false negatives from 25% with CA19-9 to 11% with the biomarker signature
Xu et al. [9]	Metabolomics Identifies Biomarker Signatures to Differentiate Pancreatic Cancer from Type 2 Diabetes Mellitus in Early Diagnosis	International Journal of Endocrinology	2021	Case-control	Seventy-six participants (26 PC patients, 27 DM patients, 23 healthy volunteers)	Metabolites detected by ultra-performance liquid chromatography coupled with tandem mass spectrometry, including: LysoPCs, FAs, peptides, and other metabolites	Identified 16 differential metabolite ions between PC patients and healthy controls, highlighting 4 (lysoPC 16:0, lysoPC 16:1, lysoPC 22:6, lysoPC 20:3) that could distinguish pancreatic cancer from DM. A biomarker signature of five metabolites (lysoPC 16:0, catelaidic acid, cerebronic acid, nonadecanetriol, asparaginyl-histidine) demonstrated high diagnostic accuracy for pancreatic cancer, with 89% sensitivity and 91% specificity. This metabolite biomarker signature outperformed CA19-9 in differentiating pancreatic cancer from DM, offering potential biomarkers for early diagnosis and differentiation from DM.
Cao et al. [10]	Potential Metabolite Biomarkers for Early Detection of Stage-I Pancreatic Ductal Adenocarcinoma	Frontiers in Oncology	2022	Prospective Cohort	Ninety serum samples (28 stage-I PDAC, 62 healthy controls) and 53 tissue samples (13 stage-I PDAC, 40 distal noncancerous tissues)	Serum and tissue metabolites, CA19-9	A 2-metabolite model (isoleucine and adrenic acid) was constructed for detecting stage-I PDAC. The model showed better diagnostic performance (AUC = 0.93 in the discovery set, 0.90 in the validation set) than CA19-9 (AUC = 0.79). The combined model of 2 metabolites and CA19-9 showed the best performance (AUC = 0.95 in the discovery set, 0.97 in the validation set).

Daemen et al. [8] conducted an experimental study published in PNAS, focusing on metabolite profiling to stratify PDAC into subtypes. They analyzed 38 samples, examining 256 metabolites and gene expression patterns. This comprehensive approach led to the identification of three distinct metabolic subtypes in PDAC: slow-proliferating, glycolytic, and lipogenic. Each subtype showed unique sensitivities to metabolic inhibitors. The glycolytic subtype demonstrated sensitivity to inhibitors of glycolysis, glutamine metabolism, and redox balance, while the lipogenic subtype was sensitive to lipid synthesis inhibitors. Interestingly, the glycolytic subtype was associated with mesenchymal features, whereas the lipogenic subtype exhibited epithelial characteristics. The researchers validated these metabolic subtypes and their sensitivities to metabolic inhibitors in approximately 200 non-PDAC cell lines, reinforcing the broader applicability of their findings.

Mayerle et al. [4] published a case-control study in the gut that aimed to develop a metabolic biomarker signature to differentiate PDAC from chronic pancreatitis (CP). This large-scale study included 914 participants: 271 with PDAC, 282 with CP, 100 with liver cirrhosis, and 261 healthy and non-pancreatic disease controls. The researchers analyzed the metabolomic profiles of 477 metabolites using advanced techniques such as gas chromatography-mass spectrometry and liquid chromatography-tandem mass spectrometry. Their final biomarker signature comprised nine metabolites plus CA19-9. This signature showed impressive diagnostic accuracy in distinguishing PDAC from CP, with area under the curve (AUC) values of 0.96 and 0.94 in the training and test sets, respectively. Notably, the biomarker signature outperformed CA19-9 alone, detecting 98% of resectable pancreatic cancer and significantly reducing both false positives and false negatives compared to CA19-9.

Xu et al. [9] conducted a case-control study published in the International Journal of Endocrinology, focusing on identifying biomarker signatures to differentiate pancreatic cancer from type 2 diabetes mellitus (DM) in early diagnosis. The study included 76 participants: 26 pancreatic cancer patients, 27 DM patients, and 23 healthy volunteers. Using ultra-performance liquid chromatography coupled with tandem mass spectrometry, they detected various metabolites, including lysophosphatidylcholines (lysoPCs), fatty acids (FAs), peptides, and other metabolites. The researchers identified 16 differential metabolite ions between pancreatic cancer patients and healthy controls, highlighting four lysoPCs (16:0, 16:1, 22:6, and 20:3) that could distinguish pancreatic cancer from DM. A biomarker signature comprising five metabolites (lysoPC 16:0, catelaidic acid, cerebronic acid, nonadecanetriol, and asparaginyl-histidine) demonstrated high diagnostic accuracy for pancreatic cancer, with 89% sensitivity and 91% specificity. This metabolite biomarker signature outperformed CA19-9 in differentiating pancreatic cancer from DM, offering the potential for early diagnosis and differentiation from DM.

Cao et al. [10] published a prospective cohort study in Frontiers in Oncology, investigating potential metabolite biomarkers for early detection of stage-I PDAC. The study analyzed 90 serum samples (28 stage-I PDAC, 62 healthy controls) and 53 tissue samples (13 stage-I PDAC, 40 distal noncancerous tissues). The researchers constructed a two-metabolite model (isoleucine and adrenic acid) for detecting stage-I PDAC. This model demonstrated superior diagnostic performance (AUC = 0.93 in the discovery set, 0.90 in the validation set) compared to CA19-9 (AUC = 0.79). Combining the two-metabolite model with CA19-9 yielded the best performance (AUC = 0.95 in the discovery set, 0.97 in the validation set), highlighting the potential of this approach for early PDAC detection.

Quality Assessment

The methodological quality of the four included studies was evaluated using standardized assessment tools appropriate for their respective study designs. The experimental study by Daemen et al. was assessed using an adapted version of the Cochrane Risk of Bias Tool, while the case-control and cohort studies were evaluated using the Newcastle-Ottawa Scale (NOS). Overall, the studies demonstrated good methodological quality, with scores ranging from 7 to 8 out of 9 stars on the NOS for the observational studies, and a rating of good quality for the experimental study (Table 2).

**Table 2:** Quality assessment table of the included studies.

Study	Study Design	Quality Assessment Tool	Score/Rating
Daemen et al. [8]	Experimental	Adapted Cochrane Risk of Bias Tool	Good quality
Mayerle et al. [4]	Case-control	Newcastle-Ottawa Scale	8/9 stars
Xu et al. [9]	Case-control	Newcastle-Ottawa Scale	7/9 stars
Cao et al. [10]	Prospective cohort	Newcastle-Ottawa Scale	8/9 stars

Daemen et al. showed strengths in its comprehensive metabolite profiling and use of diverse PDAC cell lines, with low risk of selection, detection, and reporting biases [8]. However, there was some unclear risk of performance bias due to limited details on metabolic inhibitor treatments. Mayerle et al. scored highly (8/9 stars) on the NOS, benefiting from a large sample size and validation in an independent cohort [4]. Its main limitation was the lack of reported non-response rate. Xu et al. received 7/9 stars on the NOS, with its strength lying in the comparison of PDAC with both healthy controls and diabetes patients [9]. However, it was limited by a relatively small sample size and unclear control selection process. Cao et al. scored 8/9 stars on the NOS, with notable strengths in its focus on stage-I PDAC and use of both serum and tissue samples, though it was unclear if additional factors were controlled for beyond the primary variables of interest [10].

Common limitations across studies included relatively small sample sizes (with the exception of Mayerle et al.) and potential selection bias due to single-center designs. The lack of standardization in metabolomic techniques across studies also presents a challenge for direct comparisons. Despite these limitations, the consistent findings across studies with different designs and populations strengthen the overall evidence for the potential of metabolite biomarkers in early PDAC detection. Future large-scale, multi-center studies with standardized metabolomic protocols would further enhance the quality of evidence in this field.

Discussion

This systematic review evaluates the potential of metabolite biomarkers for early detection of PDAC. Analysis of four key studies reveals promising avenues for metabolomic-based screening strategies while also highlighting challenges and areas for future research.

A significant finding across the studies is the consistent alteration of specific metabolic pathways in PDAC patients compared to healthy controls and those with benign pancreatic conditions. A biomarker signature comprising nine metabolites plus CA19-9 showed high accuracy in distinguishing PDAC from chronic pancreatitis, outperforming CA19-9 alone [4]. This signature's ability to detect 98% of resectable pancreatic cancer while reducing false positives and negatives underscores the potential of metabolite biomarkers in early PDAC detection. These findings align with other research identifying panels of metabolites associated with future pancreatic cancer risk in large prospective cohort studies [11]. Integrating metabolomic data with other molecular profiling approaches has emerged as a powerful strategy for improving diagnostic accuracy. Combining metabolite profiling with gene expression analysis can stratify PDAC into distinct metabolic subtypes with varying sensitivities to metabolic inhibitors [8]. This multi-omics approach addresses the heterogeneity of PDAC and offers a more comprehensive screening method. Integrating metabolomics with transcriptomics and proteomics provides deeper insights into PDAC biology and potential therapeutic targets [12].

Recent studies have highlighted the potential of metabolite biomarkers in differentiating PDAC from type 2 diabetes mellitus, a significant finding given the increased risk of PDAC in diabetic patients [9]. A five-metabolite signature demonstrated high diagnostic accuracy, offering a potential solution to distinguishing new-onset diabetes due to PDAC from type 2 diabetes. This work is further supported by research identifying distinct metabolic signatures in pancreatic cancer-associated diabetes, providing additional biomarkers for early detection [13].

Research on early detection of stage-I PDAC is up-and-coming. A two-metabolite model (isoleucine and adrenic acid) showed superior performance in detecting stage-I PDAC compared to CA19-9 [10]. This finding is crucial, as early detection remains the most effective strategy for improving PDAC outcomes. The importance of early detection is further emphasized by studies demonstrating that metabolomic profiling could detect pancreatic cancer up to 60 months before clinical diagnosis [14].

However, several challenges remain in translating these findings into clinical practice. The influence of confounding factors such as diet, medication, and comorbidities on metabolomic profiles presents a significant hurdle. Standardizing pre-analytical procedures and considering these confounding factors in study designs is crucial [15]. Future studies must address these confounders to ensure reproducibility and clinical applicability. Standardized workflows for metabolomics studies in epidemiology have been proposed, which could be adapted for PDAC research to improve consistency and reliability [16]. The analytical platforms used for metabolomic profiling have evolved significantly, with recent advancements in mass spectrometry techniques expanding the depth and breadth of metabolite detection. However, standardization of metabolomic methods across different laboratories remains a challenge. Consistent protocols and data reporting are needed to facilitate large-scale validation studies and clinical implementation [17,18].

Recent advancements in artificial intelligence and machine learning offer new avenues for interpreting complex metabolomic data in PDAC detection. These computational approaches have the potential to enhance the sensitivity and specificity of metabolite-based screening tools by identifying novel metabolite patterns that traditional statistical methods might overlook [19,20]. Future research should focus on validating these biomarker signatures in diverse populations, exploring their performance in high-risk individuals, and evaluating their cost-effectiveness in large-scale screening programs. Integrating metabolomics with other emerging technologies, such as liquid biopsies and circulating tumor DNA analysis, may further enhance early detection strategies for PDAC [21,22].

Additionally, investigating the role of the tumor microenvironment and its metabolic interactions could provide new insights into PDAC progression and potential therapeutic targets. Understanding metabolic reprogramming in PDAC is crucial for developing more effective diagnostic and treatment strategies [23,24]. The gut microbiome's influence on PDAC development and progression is an emerging area of research that could impact metabolite biomarker discovery. Gut microbiome modulation could enhance the efficacy of immunotherapy in PDAC, suggesting potential microbial metabolites as biomarkers [25]. This line of investigation opens up new possibilities for integrating microbiome data with metabolomics for improved PDAC detection and management.

Furthermore, the role of extracellular vesicles (EVs) in PDAC progression and their potential as a source of metabolite biomarkers is gaining attention. EVs derived from pancreatic cancer cells carry distinct metabolic signatures that could be exploited for early detection [26]. Integrating EV metabolomics with other biomarker approaches could provide a more comprehensive and sensitive screening strategy for PDAC. Lastly, the application of metabolomics in monitoring treatment response and predicting outcomes in PDAC patients is an important area for future research. Serum metabolomic profiles could predict survival in pancreatic cancer patients undergoing treatment [27]. Such approaches could not only aid in early detection but also personalized treatment strategies and prognosis assessment.

It is important to acknowledge several limitations in the current body of research. The studies reviewed had relatively small sample sizes, which may limit the generalizability of their findings. Additionally, most studies were conducted in single centers or specific populations, potentially introducing selection bias. The lack of standardization in metabolomic techniques across studies makes direct comparisons challenging. Furthermore, the influence of confounding factors such as diet, medication, and comorbidities on metabolic profiles was not consistently addressed across all studies.

Implementing metabolite-based screening strategies for PDAC could have significant economic implications. While the initial costs of developing and implementing these technologies may be substantial, the potential for earlier detection and intervention could lead to considerable long-term cost savings in healthcare. Early-stage PDAC is more amenable to curative treatments, potentially reducing the need for expensive palliative care associated with late-stage disease. However, comprehensive cost-effectiveness analyses are needed to fully understand the economic impact of these screening strategies.

To summarize, metabolite biomarkers show significant promise for the early detection of PDAC. While challenges remain, ongoing advancements in analytical technologies, bioinformatics, and our understanding of cancer metabolism continue to drive the field forward. The integration of metabolomics with other emerging technologies and multi-omics approaches offers exciting possibilities for improving PDAC detection and management. As research progresses, metabolite-based screening strategies have the potential to significantly improve early detection rates for PDAC, ultimately leading to better patient outcomes in this devastating disease. However, continued research addressing current limitations, economic considerations, and ethical implications is crucial for the successful translation of these promising findings into clinical practice.

## Conclusions

This systematic review underscores the promising potential of metabolite biomarkers for early detection of PDAC. The studies analyzed consistently demonstrate altered metabolic profiles in PDAC patients, offering new avenues for non-invasive screening strategies. Metabolite signatures, either alone or in combination with existing biomarkers like CA19-9, have shown improved diagnostic accuracy compared to traditional methods. However, several challenges remain before these findings can be translated into clinical practice. These include the need for large-scale, prospective validation studies, standardization of metabolomic methods, and consideration of confounding factors such as diet and comorbidities. The integration of metabolomics with other molecular profiling approaches and advanced technologies like artificial intelligence presents exciting opportunities for enhancing diagnostic accuracy. Future research should focus on validating these biomarker signatures in diverse populations, exploring their performance in high-risk individuals, and evaluating their cost-effectiveness in large-scale screening programs. As the field progresses, metabolite biomarkers hold the potential to significantly improve early detection rates for PDAC, ultimately leading to better patient outcomes in this devastating disease.
